# Unmet Healthcare Needs and Associated Factors in Rural and Suburban Vietnam: A Cross-Sectional Study

**DOI:** 10.3390/ijerph17176320

**Published:** 2020-08-31

**Authors:** Ju Young Kim, Dae In Kim, Hwa Yeon Park, Yuliya Pak, Phap Ngoc Hoang Tran, Truc Thanh Thai, Mai Thi Thanh Thuy, Do Van Dung

**Affiliations:** 1 Department of Family Medicine, Seoul National University Bundang Hospital, Seongnam 13620, Korea; kkamduri@snubh.org (J.Y.K.); R2374@snubh.org (D.I.K.); irisyeon7@naver.com (H.Y.P.); 2 Office of External Affairs, Seoul National University Bundang Hospital, Seongnam 13620, Korea; sunnyj@snubh.org; 3 Faculty of Public Health, University of Medicine and Pharmacy at Ho Chi Minh City, Ho Chi Minh City 72714, Vietnam; phaptran@ump.edu.vn (P.N.H.T.); truc.tt@umc.edu.vn (T.T.T.); maithanhthuy@ump.edu.vn (M.T.T.T.)

**Keywords:** health services accessibility, medically underserved area, healthcare disparities, assessment of healthcare needs

## Abstract

The purpose of this study was to examine the current utilization of healthcare services, exploring unmet healthcare needs and the associated factors among people living in rural Vietnam. This cross-sectional study was conducted with 233 participants in a rural area. The methods included face-to-face interviews using a structured questionnaire, and anthropometric and blood pressure measurements. We considered participants to have unmet health needs if they had any kind of health problem during the past 12 months for which they were unable to see a healthcare provider. Multivariate logistic regression analysis was performed to determine the factors associated with unmet healthcare needs. Of the participants, 18% (*n* = 43) had unmet healthcare needs, for reasons like transportation (30%), a lack of available doctors or medicine (47%), and communication issues with healthcare providers (16%). The multivariate logistic regression showed that living in a rural area, having stage 2 hypertension, and having insurance were associated with unmet healthcare needs. To better meet the healthcare needs in rural or suburban areas of Vietnam, allocation of adequate healthcare resources should be distributed in rural areas and insurance coverage for personalized healthcare needs might be required. Efforts should focus on availability of medicine, improvement of transportation systems, and communication skills of healthcare providers to improve access to healthcare services.

## 1. Introduction

Vietnam has made enormous progress in its socioeconomic development, including the overall health status of its citizens. It has achieved several health-related Millennium Development Goals, such as decreasing infant and maternal mortality, increased immunization coverage of 97.2% for children younger than one year, and an increased treatment rate of 80% for tuberculosis and HIV/AIDS patients [1]. Life expectancy increased from 70.5 years in 1990 to 75.8 years in 2015. In addition, there has been a rapid increase in public health facilities and availability of medical equipment due to increased funds for the health sector from both the government and private sources [2].

Still, there are several challenges and problems for Vietnam’s healthcare system. These include an emerging increase of noncommunicable diseases (NCDs), such as cardiovascular disease, cancer, and diabetes, an aging population, an inadequate capacity of the health system, and inequities in access to the healthcare system [2,3,4].

According to a study on NCD service availability in Vietnam that focuses on ethnic minorities living in a mountainous area, community health centers play a significant role in NCD care and risk factor management, but they have limited NCD preventive and treatment services, and have limited medication availability; they are also underutilized [5].

Unmet healthcare needs can be defined as the differences between the utilization of necessary healthcare services to manage a particular health problem and the actual medical service used [6,7]. Unmet healthcare needs have been reported to be associated with a high mortality rate, especially in the elderly adults population [8,9]. Unmet healthcare needs and health utilization indicators have also been used to monitor equity in health services [10,11]. Factors associated with unmet needs for healthcare services depend on the healthcare system as well as individual status, but, in general, they can be classified into three categories [12,13]. The first category is accessibility, which includes distance to medical facilities, transportation, and financial factors. The second is acceptability, which includes awareness and knowledge about healthcare. The third is availability, which includes the unavailability of certain medical services, delay, and medical services not being available in certain areas.

The purpose of this study was to examine the current utilization of healthcare services by exploring unmet healthcare needs and their associated factors among adults living in rural and suburban Vietnam.

## 2. Materials and Methods

### 2.1. Study Design and Participant Recruitment

This study was done as a part of a feasibility study in Korea’s official development assistance project, in collaboration with the University of Medicine and Pharmacy at Ho Chi Minh City. The cross-sectional study was conducted in a rural area (Binh Phuoc province), and in a suburban area (Da Lat city in Lam Dong province). These areas were selected due to cooperation from community health centers and convenience of transportation for interviewers.

Located in the Southeast region of Vietnam, Binh Phuoc, a predominantly rural province, covers an area of 6871 km^2^, and is divided into five commune-level towns, 92 communes, and 14 urban communes. The population of Binh Phuoc in 2015 was 944,400. Dak Nhau, a commune in Binh Phuoc, is in a mountainous area and is 30 to 70 km away from the district hospital. Dak Nhau commune is the residence of people belonging to an ethnic minority, especially the Stieng and Mnong people. Da Lat city, a district-level city, is located in the central highlands of Vietnam. It covers an area of 395 km^2^, spreading over 12 urban communes and four communes. The population of Da Lat in 2015 was 406,105, of which 55,596 were suburban inhabitants (13.7%). Agriculture, forestry, and handicrafts play an important part in the economy of the suburban residents. The Ta Nung commune, Tram Hanh commune, and urban commune #7 were selected. These communes are in suburban areas of Da Lat city, and are 7 to 30 km away from the district hospital.

We selected 203 participants in the Binh Phuoc province and 101 participants in Da Lat city from a list of households from the local authorities, and a total of 304 people were recruited for participation in this study. Among them, 233 were finally selected after excluding participants less than 19 years old. Well-trained researchers from the faculty of public health of the University of Medicine and Pharmacy at Ho Chi Minh City visited households with help from local health facility leaders and invited the family head or any other members of families at home to participate in the survey.

The Institutional Review Board of the University of Medicine and Pharmacy at Ho Chi Minh City reviewed and approved all activities of this study. All participants were informed about the survey and were asked for their verbal consent before collecting data. Participants could withdraw from the interview at any time without any threat or disadvantage.

### 2.2. Survey Instrument and Measurements

Face-to-face interviews were conducted using a structured questionnaire, which included questions about the participants’ socio-economic status, health problems, health service utilization, health service responsiveness with satisfaction, and required healthcare services. The socioeconomic characteristics included age, gender, marital status, education level, ethnicity, occupation, monthly income, number of family members, and health insurance. Health-related factors included self-perceived health status, smoking, drinking, physical activity, and underlying chronic diseases, such as hypertension, diabetes mellitus, dyslipidemia, heart disease, stroke, chronic lung disease, and depression. Participants were categorized as current smokers if they reported currently smoking any tobacco products such as cigarettes, cigars, or pipes. Hazardous drinking was defined as consuming at least once a month and on a single occasion more than five standard drinks [14]. Physical activity was assessed by International Physical Activity Questionnaires [15] and categorized as high level if a vigorous-intensity activity, achieving a minimum total physical activity of at least 1500 MET minutes a week, was carried out on at least 3 days, or 7 or more days of any combination of walking and moderate-intensity or vigorous-intensity activities achieving a minimum total physical activity of at least 3000 MET minutes a week. Moderate level of physical activity was defined as 3 or more days of vigorous-intensity activity and/or walking of at least 30 min per day, 5 or more days of moderate-intensity activity and/or walking of at least 30 min per day, or 5 or more days of any combination of walking and moderate-intensity or vigorous-intensity activities achieving a minimum total physical activity of at least 600 MET minutes a week.

Anthropometric measurements including height, weight, and systolic and diastolic blood pressure (SBP and DBP, mmHg) were taken by the researchers using a portable weight and height measurement device (BSM370, InBody Co., Seoul, Korea) and a blood pressure measurement device (HEM-1020, Omron Co., Tokyo, Japan). Height and weight were measured with the subjects barefoot and lightly clothed. Blood pressure was measured twice and recorded when subjects were sitting.

Body mass index was calculated as kg/m^2^. Blood pressure was chosen for the mean values of two measurements and categorized as normal (SBP < 130 and DBP < 85), prehypertension (130 ≤ SBP < 140 or 85 ≤ DBP < 90), stage 1 hypertension (130 ≤ SBP < 140 or 85 ≤ DBP < 90), or stage 2 hypertension (SBP ≥ 160 or DBP ≥ 100) [16].

Questions regarding healthcare service utilization consisted of number of admissions to the hospital or visits to an emergency department and number of visits to an outpatient clinic during the previous 12 months, and expenses during those admissions or visits.

Participants were also asked to evaluate healthcare services they had experienced, that is, how satisfied they were with the healthcare services, and were requested to suggest further improvements that it needed. Participants were compensated with a cash equivalent of USD 5 when they finished the interviews.

### 2.3. Unmet Healthcare Needs Group

Participants were asked if they had any kind of health problems during the past 12 months and whether they had access to the required healthcare providers to solve their problems. Those who had health problems and were unable to see healthcare providers were classified as being in the unmet healthcare needs group and were asked further questions to explore the reasons for not seeing healthcare providers. We classified the questions into three categories of accessibility, availability, and acceptability. For accessibility, we asked about factors such as knowledge of how to find appropriate doctors, fear, transportation, physical disabilities, difficulties in getting appointments at hospitals, language barriers, and insurance or cost issues. For availability issues, we asked about factors such as lack of availability of amenities like doctors, medicines, time, support in visiting the hospital, and health insurance. For acceptability issues, we asked about cultural/religious beliefs, communication with healthcare providers, getting enough information, treatment decision making, privacy, treatment choice, waiting time, condition of the waiting room, and hygiene status of healthcare facilities.

### 2.4. Statistical Analysis

Both descriptive and analytical statistical analyses were carried out using SAS 9.4 software (SAS Institute Inc., Cary, NC, USA). Descriptive statistical analysis was used to present the socio-demographic characteristics, healthcare service utilization, and healthcare service evaluation of participants. Student’s *t*-test and chi-squared test were used to compare the differences between the unmet healthcare needs group and the healthcare met group. Multivariate logistic regression was performed to determine the factors associated with unmet healthcare needs. The variables included into the multivariate model were selected based on individual-level predisposing, enabling, and need-related factors on access to healthcare service [17]. Predisposing factors included age, gender, education, residential area, and ethnicity. Enabling factors included having insurance, household income, marital status, number of family members, and occupation. Need for healthcare was represented by having chronic disease, self-reported health status, and previous use of healthcare utilization. The variables were included in the final model if the significance of the correlation with unmet healthcare needs was less than 0.2. The results are presented with adjusted odds ratios (OR) and 95% confidence intervals with *p*-value. The significance level was set at *p* < 0.05.

## 3. Results

Of the 233 participants, 64% were less than 50 years old and only 12% were over 65 years old. Overall, 18% of the participants (*n* = 43) had unmet healthcare needs. Sociodemographic factors of the unmet and met healthcare needs groups are presented in Table 1.

The unmet healthcare needs group had significantly higher proportions of ethnic minority people than the healthcare met group (*p* = 0.045). In addition, the number of the unmet healthcare needs group differed significantly between Binh Phuoc (35/159, 22%) and Da Lat (8/74, 11%) (*p* = 0.040), and in terms of having larger numbers of family (*p* = 0.029). The unmet healthcare needs group was more likely to have unhealthy behaviors such as smoking (*p* = 0.015) and hazardous drinking (*p* = 0.056).

Table 2 presents the healthcare utilization patterns of both groups. Higher costs with longer distance for emergency services were observed in the unmet healthcare needs group. Total costs for health services were significantly lower in the unmet healthcare needs group. The most common means of transportation was private car or motorcycle.

The unmet healthcare needs group had a higher response (30%) to the question of having no place to go for advice for health compared to the healthcare met group (5%). The most encountered health challenges in both groups were joint pain or back pain, with half of the participants reporting these conditions. Heart disease and cancer were reported significantly higher in the healthcare met group.

Table 3 shows the reasons for participants in the unmet healthcare group not using healthcare services. As for accessing healthcare services, transportation was identified as the most important factor. Regarding the availability issue, lack of available doctors and medicines each accounted for 47% of all causes. Moreover, 23% did not use insurance during the last visit, and 86% did not even receive a refund despite reporting having no problem in using insurance. Regarding the acceptability issue, communication issues with healthcare providers were the most common barrier, accounting for 16% of all causes.

Table 4 shows the factors associated with unmet healthcare needs that were identified using multivariate logistic regression analysis. Living in Binh Phuoc (OR = 3.61, 95% CI = 1.13–11.55) and having stage 2 high blood pressure (OR = 3.82, 95% CI= 1.17–12.45) were significant factors associated with unmet healthcare needs. In addition, participants who had insurance (OR = 7.11, 95% CI = 1.45–34.84) were more likely to have unmet healthcare needs.

## 4. Discussion

In this first study of unmet healthcare needs among people in rural and suburban areas of Vietnam, we identified factors related to unmet needs as well as its barriers. Unmet healthcare needs were found in 18% of participants, which was higher than 11% of the general population in Korea [18] and much higher than the unmet healthcare needs in 1.6% of the rural population in Thailand [19]. Having no healthcare resources during medical problems as well as a lack of transportation were the most common reasons among people with unmet healthcare needs. Thus, no source of care can be regarded as being potentially inaccessible to healthcare services. Further, living in a rural area as well as having insurance were significantly associated with unmet healthcare needs.

There has been a study targeting elderly Vietnamese participants for assessing their health needs [20]. The biggest for elderly Vietnamese participants was education regarding chronic disease management. In our group consisting of a relatively younger age, the most-needed services were a wellness program and health check-up. However, accessibility to healthcare services has been limited due to a lack of healthcare resources and lack of transportation systems.

How we assess people’s unmet healthcare needs is important because access to effective healthcare services is the next step in improving health for Vietnamese people living in rural areas. However, access to healthcare services is a complex concept, and measuring unmet healthcare needs could be approached on multiple levels and according to several frameworks [21]. Anderson et al. proposed three components of individual-level characteristics labeled as “predisposing factors” (demographic factors, social factors, or individual beliefs), “enabling factors” (income, health insurance, or usual source of care), and “perceived need” (perceived or evaluated need for service) [22]. These components lead to health service utilization, and this framework has produced outcomes such as appropriate utilization and consumer satisfaction. Since this study evaluated the effect of individual characteristics on unmet healthcare needs, we explored the individual variables according to Andersen’s framework.

As for predisposing factors, study participants within the unmet healthcare needs group were more likely to live in a rural area, and the group was observed to have a slightly higher proportion of ethnic monitories than within the control group. These findings are consistent with results from many studies evaluating the inequities in access to healthcare between rural and urban areas among pregnant women, hematological cancer survivors and the elderly population [23,24,25]. Several contributing factors have been recognized such as socioeconomic status within a community [26]. However, another study suggested that regional poverty is equally associated with unmet healthcare needs in both rural and urban settings [17]. Since the level of income, education, and occupation of our study participants did not show a significant difference between the unmet needs group and control group, other contextual factors such as healthcare resources or social capital might have effects on healthcare needs.

Among enabling factors, no usual source of care, having been married, and higher numbers of family members were identified as significant differences between the two groups. Insurance status has been an important factor associated with unmet healthcare needs in our study. However, having insurance seemed to be associated with unmet healthcare needs, which is contrary to other studies [27,28,29]. The coverage of health insurance increased to cover nearly 82% in 2015 in Vietnam, but financial contributions from the health insurance scheme have been accounted for in only 18% of the total health spending [1]. Thus, it is assumed that simply having insurance is not associated with healthcare needs, and it is necessary to check how insurance is specifically used and if insurance coverage is appropriate to an individual’s healthcare needs. Further, the high rate of out-of-pocket payments led to limited access to healthcare services and was possibly associated with unmet healthcare needs. In this study, we could not get data regarding how insurance was specifically covered, so the association of having insurance and unmet healthcare needs should be interpreted with caution. People with unmet healthcare needs might have an uncertain insurance coverage in finding an appropriate doctor or getting medicine when in need.

If a person has an underlying medical illness or disease, he or she has a healthcare need for the treatment. From the “need” point of view, having stage 2 high blood pressure and unhealthy health-related behaviors including smoking and hazardous drinking were observed more often in the unmet healthcare needs group. This was relatively different from previous studies, in which economic problems were significantly responsible for the accessibility problem [30,31,32]. This might be due to the relatively homogenous nature in our study population, where all of them were living in suburban or rural areas of Vietnam. Most of the participants were living on agriculture, had no formal education after primary school, had health insurance to cover medical costs, and had a monthly income that was not significantly different from the other group. In our study, personal “need” issues were important in unmet healthcare needs, and stage 2 high blood pressure could possibly be related to untreated high blood pressure issues such as cardiovascular problems or chronic kidney disease.

Nearly half of the participants could not get a prescription from healthcare providers or could not get medicine, and this was the biggest cause of unmet healthcare needs. This might reflect the importance and need for a greater investment of community-level health resource allocation such as identifying populations for whom healthcare service is potentially inaccessible, supporting them, and providing healthcare services to them [33]. More than two thirds of the study population used a motorcycle to go to the hospital, and this was also a barrier to access to healthcare services which was similar in previous published studies [23,34].

Older adults (more than 65 years old) who might have had multiple comorbidities made up only 12% of the total study participants, thus the most common health challenge people reported in our study was joint pain or back pain.

However, chronic diseases such as high blood pressure, diabetes, or chronic lung disease were also recognized as health challenges because of their economic burden, which might cause repeated, lifelong medical expenditures for the treatment of such chronic diseases [35].

There are several limitations in this study. Sites were not randomly chosen because of the availability of cooperation from commune health centers and transportation convenience for interviewers. Study participants were selected from the list of households provided by local authorities, and from this list we tried to select participants according to an even distribution of age and gender. However, our population might not be representative of people living in rural areas of Vietnam. Compared with another study using northern rural Vietnamese participants [36], our study participants seemed to be less educated (67% being less than secondary school in our study group compared with 27.4% in the northern rural group) and younger (aged more than 65 years old being 11.6% compared with 18.3% in the northern rural group). This might cause a differential effect of associated factors on unmet healthcare needs according to the different sociodemographic factors.

In our study, stage 2 high blood pressure was shown to be a strong factor associated with the unmet healthcare needs group, but this might not reflect the true hypertension status because blood pressure measurements were only taken in one day, while hypertension should be diagnosed at three or more separate days of persistent high blood pressure.

We did not link our survey results with objective data such as morbidity, mortality, health insurance status covered by national insurance, public health service provided by commune health centers, number of healthcare facilities, or number of healthcare providers including doctors and nurses. These community-based data can suggest more objective indicators including health resource allocation per capita. In addition, community-level factors associated with unmet healthcare needs were not evaluated in this study. Further research including nationally representative data and considering objective data will be necessary for establishing health policies in rural Vietnam.

Despite these limitations, this study has evaluated unmet healthcare needs among people in rural and suburban areas of Vietnam and their associated factors. Our findings show that 18% of people living in rural and suburban areas of Vietnam have unmet healthcare needs, and unavailability of medicine and transportation were barriers to accessing healthcare services. To meet the healthcare needs in rural Vietnam, allocation of healthcare resources as well as extended insurance coverage might be needed. Further studies with more representative samples of the population will be needed to evaluate unmet healthcare needs in Vietnam.

## 5. Conclusions

In conclusion, to better meet the healthcare needs of people living in rural or suburban areas of Vietnam, allocation of adequate healthcare resources should be distributed in rural areas and insurance coverage for personalized healthcare needs might be required. Efforts should focus on availability of medicine, improvement of transportation systems, and communication skills of healthcare providers to improve access to healthcare services.

## Figures and Tables

**Table 1:** Sociodemographic characteristics of research participants.

Characteristic, *n* (%) or Median (IQR)	Unmet Needs Group	Control Group	*p*-Value
	*N* = 43	*N* = 190	
Age in years			0.555
19–34	11 (26)	64 (34)	
35–49	16 (37)	59 (31)	
50–64	9 (21)	46 (24)	
≥65	7 (16)	21 (11)	
Sex			0.049
Male	25 (58)	79 (42)	
Female	18 (42)	111 (58)	
Marital status			0.192
Married	34 (79)	165 (87)	
Education levels completed			0.618
Illiterate or primary school	30 (70)	125 (66)	
Secondary or more	13 (30)	65 (34)	
Ethnicity			0.045
Vietnamese	26 (60)	131 (69)	
Mnong/Stieng	14 (33)	32 (17)	
Others	3 (7)	27 (14)	
Occupation			0.538
Farmer	32 (74)	127 (67)	
Homemaker	7 (16)	31 (16)	
Service or sale workers	3 (7)	16 (8)	
Others	1 (2)	16 (8)	
Area			0.040
Binh Phuoc	35 (81)	124 (65)	
Da Lat	8 (19)	66 (35)	
Having insurance			0.132
Yes	34 (79)	128 (67)	
No	9 (21)	62 (33)	
Number of family members (median, IQR)	5 (4, 6)	4 (3, 5)	0.029
Monthly income, VND 1000 (median, IQR)	3333 (1666, 6000)	3500 (1500, 5000)	0.930
BMI, kg/m^2^			0.442
<18.5	20 (47)	112 (60)	
18.5–22	9 (21)	27 (14)	
23–24	9 (21)	29 (15)	
≥25	5 (12)	20 (11)	
Blood pressure, mmHg			0.054
SBP < 130 and DBP < 85	16 (37)	108 (57)	
130 ≤ SBP < 140 or 85 ≤ DBP < 90	9 (21)	36 (19)	
140 ≤ SBP < 160 or 90 ≤ DBP < 100	10 (23)	31 (16)	
SBP ≥ 160 or DBP ≥ 100	8 (19)	15 (8)	
Chronic disease			
Hypertension	7 (16)	33 (17)	
Diabetes mellitus	1 (2)	7 (4)	
Dyslipidemia	0 (0)	10 (5)	
Heart disease	1 (2)	27 (14)	
Stroke	0 (0)	4 (2)	
Chronic lung disease	5 (12)	7 (4)	
Chronic viral hepatitis	1 (2)	1 (1)	
Depression	4 (9)	37 (19)	
Currently smoking			0.015
Yes	18 (42)	45 (24)	
Hazardous drinking			0.056
Yes	15 (35)	38 (20)	
Physical activity			0.127
High	12 (28)	55 (29)	
Moderate	6 (14)	52 (27)	
Low	25 (58)	80 (42)	

BMI: body mass index; SBP: systolic blood pressure; DBP: diastolic blood pressure, VND: Vietnam dong, IQR: interquartile range.

**Table 2:** Healthcare utilization and healthcare service needs.

Characteristic, *n* (%) or Median (IQR)	Unmet Group	Control Group	*p*-Value
	*N* = 43	*N* = 190	
Number of admissions to the hospital during the past 12 months	0	1	
Average cost per night at the hospital (VND 1000)	Not applicable	300	
Distance from home to the hospital (km)	Not applicable	33	
Number of visits to emergency room	5	26	0.720
Average costs per emergency service at the hospital (VND 1000)	1300 (1000, 1700)	1000 (500, 1300)	0.515
Distance to the emergency department (km)	34 (30, 37)	25 (4, 38)	0.434
Total cost for health services during the past 12 months (VND 1000)	15 (0, 750)	1500 (300, 3000)	<0.001
Having a place to go for advice about health	30 (70)	180 (95)	<0.001
Type of transportation			0.348
Private car or motorcycle	28 (70)	141 (74)	
Public car	1 (3)	5 (3)	
Ambulance	1 (3)	2 (1)	
Other	6 (15)	36 (19)	
Transportation time in minutes	20 (10, 90)	30 (15, 90)	0.557
General health status			0.932
Excellent/Very good	1 (2)	5 (3)	
Good	9 (21)	43 (23)	
Fair	21 (49)	98 (52)	
Poor	12 (28)	44 (23)	
Health status compared to the previous 12 months			0.142
Better	7 (16)	20 (11)	
Worse	19 (44)	115 (61)	
About the same	17 (40)	55 (29)	
Place to go to when you are sick			0.086
District hospital	13 (30)	55 (29)	
Emergency department	8 (19)	24 (13)	
Private Health Clinic	7 (16)	64 (34)	
Commune health Center	6 (14)	58 (31)	
University/teaching hospital	3 (7)	17 (9)	
Traditional	1 (2)	3 (2)	
Others	6 (14)	16 (8)	
Top three health challenges you are dealing with			
Joint pain or back pain	21 (49)	94 (49)	0.939
Chronic disease	7 (16)	55 (29)	0.089
Stroke	6 (14)	20 (11)	0.519
Cancer	5 (12)	51 (27)	0.035
Heart disease	4 (9)	50 (26)	0.017
What is needed to improve the health of you, your family, and neighbors			
Wellness services	19 (44)	81 (43)	0.853
Health screening program	15 (35)	68 (36)	0.911
Healthier food	10 (23)	56 (29)	0.413
Specialized physicians	8 (19)	64 (34)	0.053
Types of health screenings and services needed			
Routine medical check-up program	18 (42)	93 (49)	0.401
Blood pressure management	11 (26)	72 (38)	0.128
Disease outbreak prevention	11 (26)	35 (19)	0.328
Treatment of alcohol abuse	10 (23)	18 (9)	0.012

VND: Vietnam dong, IQR: interquartile range.

**Table 3:** Reasons for not using health services in the unmet healthcare needs group.

Reasons	Prevalence *n* (%)
**Accessibility**	
Transportation	13 (30)
Do not know how to find doctors	5 (12)
No insurance and unable to pay for the care	4 (9)
Unable to pay co-pays/deductibles	4 (9)
Fear	3 (7)
Language barriers	2 (5)
**Availability**	
Healthcare provider did not prescribe any medicine at your last visit	20 (47)
Unable to get medicines prescribed	20 (47)
Did not use healthcare insurance at last visit	10 (23)
Lack of availability of doctors	6 (14)
Unable to receive refund from healthcare insurance	4 (9)
**Acceptability**	
Clarity of communication	7 (16)
Waiting room	5 (12)
Health services in general	5 (12)
Information about treatment	4 (9)
Involvement in decision making	4 (9)
Room hygiene at health facilities	4 (9)
Do not understand the need to see a doctor	3 (7)
Time healthcare provider spent for you	3 (7)
Freedom to choose healthcare provider	1 (2)

**Table 4:** Factors associated with unmet healthcare needs group.

Factors	Odds Ratio	95% Confidence Interval	*p*-Value
Age (in years)			
19–34	1.00	1.00	
35–49	2.71	0.62–11.78	0.863
50–64	1.49	0.41–5.39	0.548
≥65	2.69	0.51–14.24	0.545
Sex			
Female	0.47	0.19–1.11	0.085
Marital status			
Married	0.31	0.10–1.08	0.062
Education levels completed			
Illiterate or primary school	1.00	1.00	
Secondary or more	2.24	0.82–6.10	0.115
Ethnicity			
Vietnamese	1.00	1.00	
Ethnic minorities	2.52	0.83–7.62	0.101
Occupation			
Farmer	1.00	1.00	
Others	0.54	0.19–1.56	0.257
Area			
Da Lat	1.00	1.00	
Binh Phuoc	3.61	1.13–11.55	0.031
Having insurance			
No	1.00	1.00	
Yes	7.11	1.45–34.84	0.016
Number of family members increase by one person	1.07	0.76–1.50	0.070
Blood pressure, mmHg			
SBP < 130 and DBP < 85	1.00	1.00	
130 ≤ SBP < 140 or 85 ≤ DBP < 90	1.93	0.64–5.81	0.856
140 ≤ SBP < 160 or 90 ≤ DBP < 100	2.58	0.79–8.34	0.373
SBP ≥ 160 or DBP ≥ 100	3.82	1.17–12.45	0.037

BMI: body mass index; SBP: systolic blood pressure; DBP: diastolic blood pressure.
